# A multiplex qPCR approach for detection o*f pfhrp2* an*d pfhrp3* gene deletions in multiple strain infections of *Plasmodium falciparum*

**DOI:** 10.1038/s41598-019-49389-2

**Published:** 2019-09-11

**Authors:** Tobias Schindler, Anna C. Deal, Martina Fink, Etienne Guirou, Kara A. Moser, Solomon M. Mwakasungula, Michael G. Mihayo, Said A. Jongo, Prosper P. Chaki, Salim Abdulla, Paulo C. Manrique Valverde, Katherine Torres, Jose R. Bijeri, Joana C. Silva, Stephen L. Hoffman, Dionicia Gamboa, Marcel Tanner, Claudia Daubenberger

**Affiliations:** 1 Department of Medical Parasitology and Infection Biology, Swiss Tropical and Public Health Institute, Basel, Switzerland; 2 University of Basel, Basel, Switzerland; 3 Institute for Genome Sciences, University of Maryland School of Medicine, Baltimore, Maryland USA; 4 Bagamoyo Research and Training Centre, Ifakara Health Institute, Bagamoyo, United Republic of Tanzania; 5 Laboratorios de Investigacion y Desarrollo, Facultad de Ciencias y Filosofia & Instituto de Medicina Tropical, Alexander von Humboldt Universidad Peruana Cayetano Heredia, Lima, Peru; 6 Equatorial Guinea Malaria Vaccine Initiative, Malabo, Equatorial Guinea; 7 Sanaria Inc., Rockville, Maryland USA

**Keywords:** Malaria, Infectious-disease diagnostics, High-throughput screening

## Abstract

The rapid and accurate diagnosis of *Plasmodium falciparum* malaria infection is an essential factor in malaria control. Currently, malaria diagnosis in the field depends heavily on using rapid diagnostic tests (RDTs) many of which detect circulating parasite-derived histidine-rich protein 2 antigen (PfHRP2) in capillary blood. *P*. *falciparum* strains lacking PfHRP2, due to *pfhrp2* gene deletions, are an emerging threat to malaria control programs. The novel assay described here, named qHRP2/3-del, is well suited for high-throughput screening of *P*. *falciparum* isolates to identify these gene deletions. The qHRP2/3-del assay identified *pfhrp2* and *pfhrp3* deletion status correctly in 93.4% of samples with parasitemia levels higher than 5 parasites/µL when compared to nested PCR. The qHRP2/3-del assay can correctly identify *pfhrp2* and *pfhrp3* gene deletions in multiple strain co-infections, particularly prevalent in Sub-Saharan countries. Deployment of this qHRP2/3-del assay will provide rapid insight into the prevalence and potential spread of *P*. *falciparum* isolates that escape surveillance by RDTs.

## Introduction

Malaria is an infectious disease with an estimated 219 million cases globally and was responsible for 435’000 deaths in 2017. More than 90% of these malaria cases and deaths occurred in sub-Saharan Africa with *Plasmodium falciparum* as the most pathogenic malaria parasite species, accounting for the vast majority of clinical malaria cases^1^.

Advances have been made in malaria control which have contributed to the decline in malaria prevalence observed worldwide with improved diagnostic tests and better access to malaria treatment contributing significantly to this development^1^. The rapid and accurate diagnosis and treatment of malaria cases is an essential factor in the control of malaria. Rapid diagnostic tests (RDTs) are becoming the most widely used method to diagnose malaria infections in the field with 245 million RDTs distributed worldwide in 2017^1^. In sub-Saharan Africa an estimated 75% of malaria tests conducted in 2017 were based on RDTs^1^. Malaria RDTs are based on an immuno-chromatographic assay using a lateral-flow device which allows the detection of malaria antigens in usually 5 to 15 µL of capillary blood^2^. RDTs provide results within 20 minutes and can be employed by inexperienced health workers operating in resource-limited settings^3^. RDTs recognizing circulating histidine-rich protein 2 (PfHRP2) for sensitive and specific detection of *P*. *falciparum* make up more than 90% of RDTs currently in use^4^. The relatively high abundance and stability of PfHRP2 in the blood of infected patients and expression by *P*. *falciparum* during the erythrocytic stage makes this antigen a valuable biomarker for malaria infection^5^. PfHRP3, a protein also expressed by *P*. *falciparum* with high level of structural similarity to PfHRP2, might be also recognized by some of the monoclonal antibodies used in the RDTs^6^. RDTs are critical diagnostic tools for identifying symptomatic malaria infections; however, due to the reduced performance in infections with low parasite density, its use for the diagnosis of malaria infection in asymptomatic individuals is rather limited^7^.

Recent studies report on reduced diagnostic performance of PfHRP2-based RDTs which were attributed to genetic diversity of the *pfhrp2/3* genes^6^, differences in expression level of PfHRP2/3 antigen in parasite field strains^8^ or isolates lacking *pfhrp2* and/or *pfhrp3* genes^9^. *P*. *falciparum* isolates lacking *pfhrp2* and/or *pfhrp3* genes are found around the world, with different proportions of the circulating *P*. *falciparum* population affected. The regions with the highest proportions of *P*. *falciparum* strains carrying *pfhrp2* deletions are South America and sub-Saharan Africa^10^. Since malaria control programmes depend on reliable diagnosis of malaria cases using RDTs, parasites lacking *pfhrp2/3* genes pose a threat to malaria control and local elimination efforts^11^.

The presence or absence of *pfhrp2/3* genes is usually determined by amplifying these genes by polymerase chain reaction (PCR). Several different (nested) PCR protocols have been published and a deletion is reported if there is no amplification of the *pfhrp2/3* genes in the presence of an amplification in at least two *P*. *falciparum* single copy genes^12^. The conventional nested PCR methods are time consuming, requiring separate reactions for each target gene amplification as well as gel electrophoresis for visualization of the PCR products. Additionally, there are methodological issues related to this approach which assumes identical PCR performance of the *pfhrp2/3* and the reference genes. Particularly at lower parasitemia levels with a small number of DNA target molecules present, unavoidable stochastic effects can play a major role and might lead to false reporting of *pfhrp2/3* deletions. Furthermore, none of the published methods detecting *pfhrp2/3* deletions can identify “masked” deletions in multiple strain infections with only one out of several *P*. *falciparum* strains carrying a *pfhrp2* and/or *pfhrp3* gene deletion^13^. These limitations of recommended molecular monitoring methods could result in an underestimation of the prevalence of *P*. *falciparum* strains with *pfhrp2/3* deletions, especially in regions with high proportions of multiple strain co-infections.

This paper presents a novel, quantitative PCR-based method for detecting *pfhrp2* and *pfhrp3* gene deletions suitable for high throughput screening of *P*. *falciparum* isolates. The qHRP2/3-del (quantitative detection of *pfhrp2* and *pfhrp3* deletion) assay was developed as a multiplex assay, with the ability to amplify individually and specifically the *pfhrp2* and *pfhrp3* genes together with a single copy gene, the *P*. *falciparum* ribonucleotide reductase R2_e2 (*pfrnr2e2*)^14^, as an internal reference. The quantitative nature of the qHRP2/3-del assay provides the basis for estimating the proportions of *P*. *falciparum* strains carrying *pfhrp2* and *pfhrp3* deletions in regions with multi-clonal malaria infections.

## Results

### Design and evaluation of the novel qHRP2/3-del assay

We aimed at improving the detection of *pfhrp2* and *pfhrp3* gene deletions by developing a quantitative PCR-based assay able to detect and quantify *pfhrp2* and *pfhrp3* genes in a single reaction. Given the high nucleotide sequence similarity and the repetitive structure of the *pfhrp2* and *pfhrp3* genes, nucleotide regions serving as targets for primers and probes were limited (Supplementary File 1). The primer and probe combinations selected for our assay (Table 1) bind to a region spanning exon 1 and exon 2 of both genes. Absence of amplification will therefore indicate a deletion of the entire genes or partial gene deletions including exon 1, the intron and first 96 base pairs of exon 2. Although there are chromosome breaking points outside the amplified regions, in particular the section that contains the repeats and epitopes detected by RDTs, analysis of field isolates suggest that the selected regions are highly predictive for *pfhrp2/3* deletions in field strains^9,12,15,16^.

**Table 1:** Oligonucleotide sequences used for qHRP2/3-del assay.

Target gene	Size	Oligo name	Oligo sequence [5′ to 3′]	Fluorophores	Conc. in 5 × PrimerMix^a^
*pfrnr2e2* (PF3D7_1015800)	107 bp	IC-PfRNR2E2 fwd	AGTATCCAAAACACTATAATTCCAAGTAC	—	1.5 µM
		IC- PfRNR2E2 rev	ATTTTCTCCTTTCTTAACAGTTTCTTCC	—	1.5 µM
		IC-PfRNR2E2 Cy5	CCTTTTAGTTGGCCCGAATTTACAA	Cy5-BHQ2	1.125 µM
*pfhrp2* (PF3D7_0831800)	286 bp	PfHRP2 fwd^b^	GTATTATCCGCTGCCGTTTTTGCC	—	1.5 µM
		PfHRP2 rev^b^	TCTACATGTGCTTGAGTTTCG	—	1.5 µM
		PfHRP2 TxRd	TTCCGCATTTAATAATAACTTGTGTAGC	TexasRed-BHQ2	0.375 µM
*pfhrp3* (PF3D7_1372200)	289 bp	PfHRP3 fwd	ATATTATCCGCTGCCGTTTTTGCT	—	1.5 µM
		PfHRP3 rev	CCTGCATGTGCTTGACTTTCGT	—	1.5 µM
		PfHRP3 YY	CTCCGAATTTAACAATAACTTGTTTAGC	YakimaYellow-BHQ2	0.75 µM

^a^All oligonucleotides are premixed as a 5× primer mix.

^b^Oligonucleotide sequences obtained from Abdallah *et al*.^32^.

We designed a multiplex qPCR assay using three differently labelled TaqMan assays detecting the *pfhrp2* (PF3D7_0831800) and *pfhrp3* (PF3D7_1372200) genes with the single copy gene *pfrnr2e2* (PF3D7_1015800) as the internal control. The sequence alignment of the *pfhrp2* and *pfhrp3* genes highlighting the oligo binding regions is shown in Supplementary File 1.

**Figure 1:**
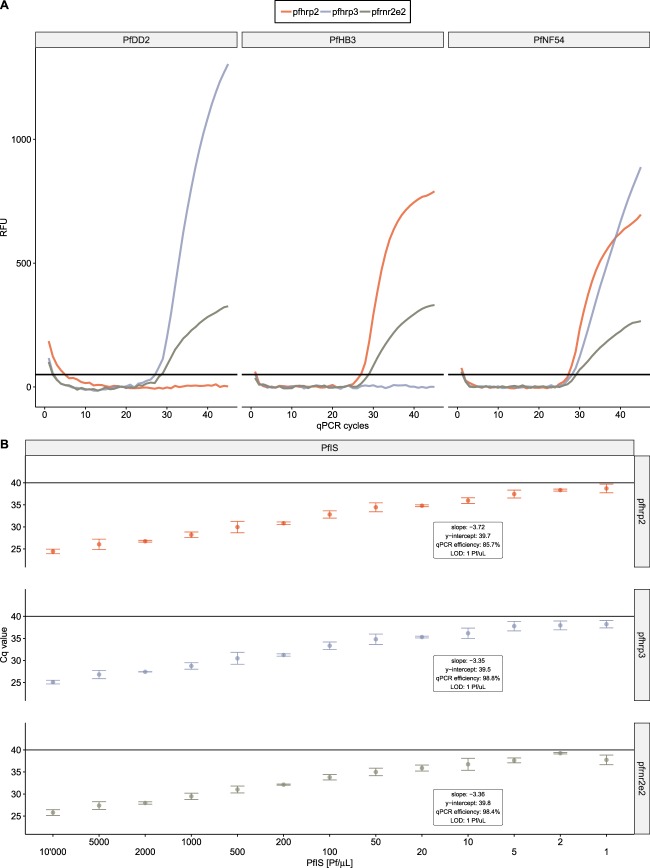
Multiplex detection of *pfhrp2* and *pfhrp3* genes using the qHRP2/3-del assay. (**A**) The qHRP2/3-del assay amplifies *pfhrp2*, *pfhrp3* and *pfrnr2e2* target sequences in a multiplex qPCR reaction and correctly identifies strains carrying either a *pfhrp2* deletion (PfDD2), a *pfhrp3* deletion (PfHB3) or no deletion (PfNF54). (**B**) Performance characteristic of each individual amplification assay, run within the multiplex qHRP2/3-del assay, is shown. Correlation with high linearity between serially diluted WHO international standard for *P*. *falciparum* NATs (PfIS) and Cq values was obtained and used to calculate the qPCR efficiency. Cq values above 40 (black line) are considered negative.

The multiplexed assays correctly identify *P*. *falciparum* strains carrying known deletions of *pfhrp2* (PfDD2 strain) and *pfhrp3* (PfHB3 strain) as well as a strain without deletion (PfNF54 strain) (Fig. 1A). The multiplexed assays show comparable characteristics in terms of sensitivity and qPCR performance. Using DNA extracted from cultured parasites, all three targets are detected in samples with parasitemia as low as 1 parasite/µL and an inverse linear correlation between Cq values and parasite densities ranging from 1 to 10’000 parasites/µL was observed. The qPCR efficiencies were calculated as 85.7%, 98.8% and 98.4% for the amplification of *pfhrp2*, *pfhrp3* and *pfrnr2e2*, respectively (Fig. 1B). The qHRP2/3-del assay was next tested using purified DNA from eight culture adapted *P*. *falciparum* strains from Africa (Pf3D7, PfNF54, PfNF166.C8), South and Central America (Pf7G8, PfHB3), South East Asia (PfNF135.C10, PfDD2) and Papua New Guinea (PfFC27) with known deletion status of the *pfhrp2* and *pfhrp3* genes. The Cq values for amplification of *pfrnr2e2* were comparable between the eight strains amplified and no significant differences of Cq values for the *pfhrp2* gene and *pfhrp3* gene across the strains carrying the genes was observed. Sequence alignments of PfNF135.C10, Pf3D7, Pf7G8, PfNF54 and PfNF166.C8 did not reveal sequence variation in the oligo binding regions of *pfhrp2* (Supplementary File 2) or *pfhrp3* (Supplementary File 3) supporting these findings. DNA derived from five non-*falciparum Plasmodium* species (*P*. *ovale curtisi*, *P*. *ovale wallikeri*, *P*. *malariae*, *P*. *knowlesi*, *P*. *vivax*) was tested with the qHRP2/3-del assay and did not result in amplification of any target demonstrating the specificity for *P*. *falciparum*. In summary, we developed a *P*. *falciparum*-specific multiplex qPCR assay that allowed the simultaneous amplification of the *pfhrp2*, *pfhrp3* and *pfrnr2e2* genes in a single reaction with high efficiency and ability to correctly identify *pfhrp2* and *pfhrp3* gene deletions.

### Analysis of *P*. *falciparum* field strains with qHRP2/3-del assay

**Figure 2:**
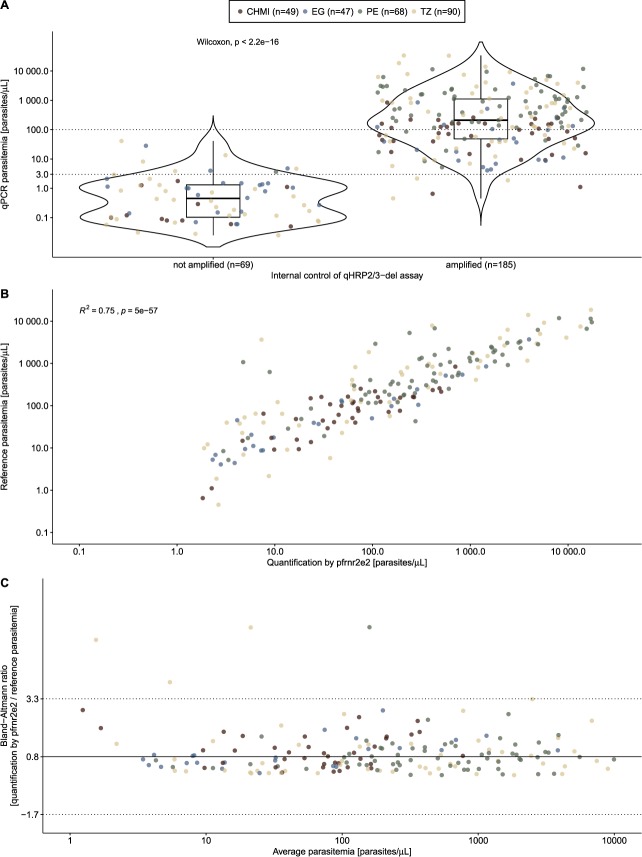
Detection and quantification of field samples using qHRP2/3-del assay. (**A**) Amplification rate of *pfrnr2e2* target, the internal control of qHRP2/3-del assay and association with parasitemia levels. Wilcoxon-Mann-Whitney test was used for comparison of parasitemia between groups. The dashed line at 3 parasites/µL represents the parasitemia at which more than 95% of the samples were amplified, while the dashed line at 100 parasites/µL represents the LOD of RDTs at which all samples are successfully amplified. (**B**) Correlation of parasitemia levels, obtained from diagnostic qPCR assays, and parasitemia, derived from the internal control of qHRP2/3-del assay, is shown. The color represents the different sample sets and R^2^ the Spearman’s rank correlation coefficient. (**C**) Bland-Altman plot of average parasitemia (x-axis) and ratio of parasitemia levels calculated between internal control of qHRP2/3-del assay and diagnostic qPCR assays (y-axis). Average ratio (black line) and 95% limits of agreement (dashed line) are depicted.

The qHRP2/3-del assay was next tested using a collection of 254 *P*. *falciparum* isolates originating from East Africa, Central-West Africa and Latin America (Table 2). The infection status and parasitemia levels were well established in these samples by using published diagnostic qPCR assays routinely used in the laboratories in Tanzania^17^, Equatorial Guinea^18^ and Peru^19^. The overall median parasitemia in these samples was 75.7 parasites/µL (IQR: 2.2–571.6), which is below the LOD of 100 parasites/µL for PfHRP2-based RDTs^20,21^. First, the ability of the *pfrnr2e2* singly copy gene to serve as internal assay control and to quantify parasitemia levels was assessed. Out of the 254 samples, 186 (73.2%) amplified the *pfrnr2e2* singly copy gene. Failure in amplification of *pfrnr2e2* was associated with low parasitemia levels (Fig. 2A). In samples with parasitemia levels of 3 parasites/µL and above, more than 95% of all samples were amplified successfully. In samples with parasitemia >100 parasites/µL, the lower limit of detection for PfHRP2-based RDTs, all qPCR reactions were positive for *pfrnr2e2*. Parasitemia levels determined by using the amplification of *pfrnr2e2* correlated closely with parasite densities obtained from *P*. *falciparum* diagnostic qPCR assays (Fig. 2B), this is supported by the findings of the Bland-Altman plot which demonstrates a high order of agreement (Fig. 2C). The average ratio of parasite quantification based on diagnostic qPCR assays and qHRP2/3-del assay is 0.8 (95% CI: −1.7–3.3). In summary, the qHRP2/3-del assay amplifies 95% of samples with parasitemia levels of 3 parasites/µL and above and can be used to reliably quantify parasite levels in field samples.

**Table 2:** Field samples used for evaluation of qHRP2/3-del assay.

Sample set	Description of sample set	Number of *P*. *falciparum* positive samples^b^	Parasitemia in parasites/µL (Median/IQR)	Amplification rate by qHRP2/3-del assay^c^
CHMI^a^	CHMI in TZ with PfNF54 strain (no deletion)	49	51.1 (1.5–152.5)	78%
PE	Peruvian samples around Iquitos city. High proportion of *pfhrp2/3* deletions	68	592.4 (186.7–1982.0)	99%
EG	Blood donors with asymptomatic malaria infection living on Bioko Island, Equatorial Guinea	47	4.8 (1.0–45.3)	51%
TZ	Sampling of symptomatic volunteers at two health facilities in Southern Tanzania	90	38.8 (0.7–808.6)	62%
Combined		**254**	**75.7 (2.2–571.6)**	**73%**

^a^Controlled Human Malaria Infection.

^b^All confirmed by diagnostic qPCR assays.

^c^Positive for internal control of assay (*pfrnr2e2*).

### Identification of *pfhrp2* and *pfhrp3* gene deletions using qHRP2/3-del assay

**Figure 3:**
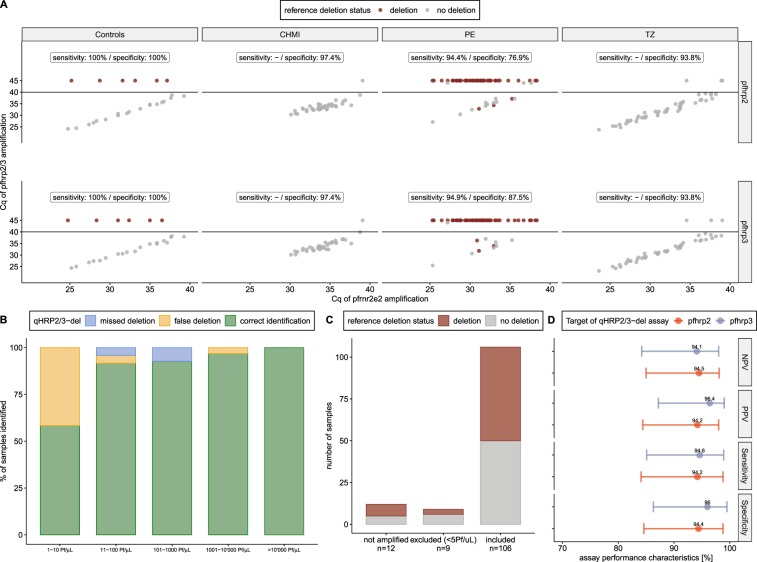
Diagnostic performance of qHRP2/3-del assay. (**A**) Samples with Cq values for *pfhrp2* and *pfhrp3* amplification >40 (shown on y-axis, black line indicates cut-off) are considered to carry a *pfhrp2/3* deletion. Reference deletion status, based on nested PCR, is color coded (red = deletion, grey = no deletion). (**B**) Proportion of correctly and incorrectly identified *pfhrp2/3* deletion status grouped by parasitemia. (**C**) Number of samples included for analysis by qHRP2/3-del assay (n = 106), excluded due to ultra-low parasitemia (n = 9) and not amplified (n = 12). (**D**) Analytical validation of qHRP2/3-del assay performance was assessed by comparing it to nested PCR. Standard parameters such as sensitivity, specificity, positive predictive value (PPV), and negative predictive value (NPV) including their 95% confidence intervals are shown.

Next, we wanted to establish the performance of the qHRP2/3-del assay in comparison with nested PCR. Samples with known *pfhrp2/3* deletion status obtained from four different sources were included. Serial dilutions of DNA purified from PfDD2 (*pfhrp2* deletion), PfHB3 (*pfhrp3* deletion) and PfIS (no deletion) served as controls. Samples from CHMI using PfNF54 (no deletion) were added to test the specificity of the qHRP2/3-del assay. Two sample sets genotyped by nested PCR, one from Tanzania (TZ) dominated by *P*. *falciparum* strains without deletions and one from Peru (PE), with a high proportion of *pfhrp2/3* deletions were analysed. The Peruvian sample set consisted of 54 samples with both genes deleted and 7 samples with only one gene deleted. The qHRP2/3-del assay defines a deletion as failure of amplification of the *pfhrp2/3* genes (Fig. 3A, y axis, Cq set to 45) in samples which are positive for the internal control, *pfrnr2e2* (Fig. 3A, x axis). Sensitivity is defined as the proportion of correctly identified *pfhrp2/3* deletions, while specificity is the proportion of correctly identified strains without *pfhrp2/3* deletions. All control samples with known deletion status were identified as expected (Fig. 3A, first panel). Importantly, the qHRP2/3-del assay correctly identified samples with parasitemia levels ranging from 1–10’0000 parasites/µL, demonstrating the dynamic range of at least 5 logs of this assay. In samples collected from volunteers that have undergone CHMI with PfNF54 (CHMI, n = 38), one sample that is positive for *pfhrp2/3* genes was wrongly detected as a double deleted parasite, resulting in a reduced specificity (Fig. 3A, second panel). A high sensitivity was achieved with the Peruvian samples (PE, n = 67), *pfhrp2* and *pfhrp3* deletions were detected with sensitivity of 94.4% and 94.9%, respectively (Fig. 3A, third panel). The low specificity of 76.9% and 87.5% for *pfhrp2* and *pfhrp3*, respectively, is based on the incorrect detection of deletions in three samples. Among the samples from Tanzania (TZ, n = 56), no *pfhrp2/3* deletions were detected by the nested PCR. In contrast, the qHRP2/3-del assay identified three deletions, resulting in a specificity of 93.8% (Fig. 3A, fourth panel).

Grouping the samples with missed deletions (reducing the sensitivity) and the false deletions (reducing the specificity) by parasitemia levels revealed a high proportion of false deletions among the samples with the lowest parasitemia levels (Fig. 3B). Based on these findings, the inclusion criteria for samples to be analysed by qHRP2/3-del assay was changed. The threshold for the *pfrnr2e2* gene amplification was reduced from Cq < 40 to Cq < 37.5, corresponding to parasitemia levels of 5 parasites/µL. Based on these new inclusion criteria, the qHRP2/3-del assay obtained results from 106 samples out of 127 samples (inclusion rate of 83.5%) (Fig. 3C). 12 samples were not amplified by the qHRP2/3-del assay and an additional 9 samples excluded based on the new inclusion criteria. Samples which were not amplified by the qHRP2/3-del assay were mainly ultra-low parasite density samples from Tanzania (11 out of 12).

In 99 out of 106 samples (93.4%), the *pfhrp2/3* deletion status was identical when compared between qHRP2/3-del assay and nested PCR. This is reflected in the near perfect agreement between these two PCR based diagnostic methods for each of the amplified targets. Cohen’s kappa was calculated as 0.89 and 0.91 for *pfhrp2* and *pfhrp3*, respectively. Out of the seven samples which were misidentified in four samples both *pfhrp* genes were affected, while in two samples the *pfhrp2* and in one sample the *pfhrp3* status was misclassified. For four misidentified samples with higher parasitemia levels the possibility of sample mix-up or cross-contamination cannot be excluded, since these samples were located next to each other on the DNA plate which was shipped. In summary, the qHRP2/3-del assay specificity (94.4% and 96.0% for *pfhrp2* and *pfhrp3*, respectively) and sensitivity (94.2% and 94.6% for *pfhrp2* and *pfhrp3*, respectively) were above 90%. The negative predictive value (NPV) was calculated as 94.5% and 94.1% and the positive predictive value (PPV) as 94.2% and 96.4%, for *pfhrp2* and *pfhrp3*, respectively (Fig. 3D).

### Multiple strain *P*. *falciparum* infections are masking *pfhrp2* and *pfhrp3* deletions

**Figure 4:**
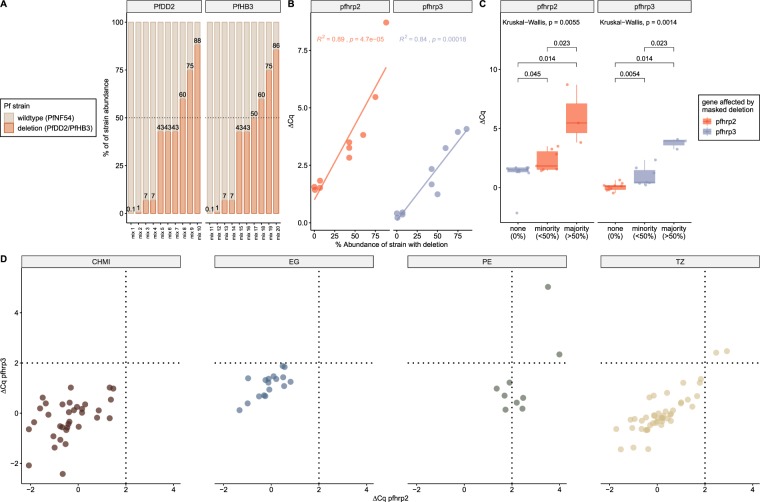
Identification of masked *pfhrp2/3* deletions in multiple strain infections. (**A**) Mixtures containing two strains, one with a *pfhrp* deletion (PfDD2 or PfHB3) and no deletion (PfNF54), were generated. (**B**) Correlation between abundance of strain carrying deletion and ΔCq is shown for both targets, *pfhrp2* (red) and *pfhrp3* (blue). (**C**) The ΔCq approach distinguishes between strain mixtures not carrying deletions, mixtures with minority abundance as well as majority abundance of strains with deletions. Statistical comparison was performed using the Kruskal-Wallis test followed by Wilcoxon-Mann-Whitney for pairwise comparisons. (**D**) The ΔCq approach of the qHRP2/3-del assay was applied to four sample collections to identify “masked” *pfhrp2/3* deletions. The control group, based on samples from CHMI, did not reveal isolates with increased ΔCq values. The dashed lines represent the ΔCq cut-off values for *pfhrp2* (x-axis) and *pfhrp3* (y-axis).

In many malaria endemic regions, particularly in sub-Saharan Africa, infections with multiple strains of *P*. *falciparum* are common^22^. A blood sample carrying multiple *P*. *falciparum* strains with and without *pfhrp2/3* deletions will result in failure to detect the deletion by nested PCR if the parasitemia level of the strain without deletion is sufficiently high for amplification. This limitation leads most likely to an underestimation of the prevalence of *pfhrp2/3* gene deletions in regions with high prevalence of multiple strain infections. We reasoned that the qHRP2/3-del assay could offer a solution by calculating the difference between the Cq values obtained for amplification of *pfhrp2* or *pfhrp3* and *pfrnr2e2*. To demonstrate the ability of the qHRP2/3-del assay to correctly identify and quantify “hidden” or “masked” *pfhrp2/3* gene deletions in mixed infections, we first tested defined mixtures of DNA from PfNF54 (no *pfhrp2/3* deletions) and PfDD2 (*pfhrp2* deletion) or PfHB3 *(pfhrp3* deletion) in a range of different ratios. For each combination of strain mixtures, PfDD2/PfNF54 or PfHB3/PfNF54, 10 mixtures were prepared containing varying ratios of strains with and without a *pfhrp2/3* deletion (Fig. 4A). The contribution from PfDD2 and PfHB3 strains to these mixtures ranged from 0.1% to 88% and 0.1% to 86%, respectively. In seven mixtures, the strain with a deletion constituted the minority (with less than 50% abundance) and in three mixtures the majority (with more than 50% abundance). None of these mixtures failed to amplify the *pfhrp2/3* genes, even if the strain carrying the deletion constituted the majority in the mixture. A positive correlation between abundance of isolate carrying a deletion and an increase of ΔCq (Cq of *pfhrp2* or *pfhrp3* minus Cq of *pfrnr2e2*) is observed (Fig. 4B). The qHRP2/3-del assay does not only successfully identify “masked” *pfhrp2/3* deletions but can also discriminates between mixtures where the strain with the deletion constitutes the majority or minority (Fig. 4C). A ΔCq cut-off value of 2.0 was chosen to identify “masked” *pfhrp* gene deletions. Applying this cut-off to our sample collections revealed that two isolates each from Tanzania and Peru have high ΔCq values for both *pfhrp* genes indicative of the presence of “masked” *pfhrp2/3* deletions (Fig. 4D). Three additional samples from the Peruvian collection had a ΔCq value > 2 for the *pfhrp2* gene only. No ΔCq values above 2 were found in Equatorial Guinean isolates and among samples collected from volunteers undergoing CHMI (Fig. 4D). These experiments demonstrate that by calculating the ΔCq values between Cq for *pfrnr2e2* and *pfhrp2* or *pfhrp3*, “masked” deletions can be identified.

## Discussion

*P*. *falciparum* strains carrying *pfhrp2/3* deletions are an emerging threat to malaria control and elimination programs around the world. Novel analysis tools enabling high-throughput screening of *P*. *falciparum* populations from the field are needed. The currently published methods, mostly based on nested PCR, have clear limitations in that these methods are extremely time consuming, prone to detection of incorrect deletions at low parasitemia levels and unable to identify “masked” deletions in multiple strain co-infections.

The sensitivity and specificity of the PfHRP2/3-del assay is comparable to the widely used nested PCR. However, the novel qHRP2/3-del assay is well suited for high throughput screening of *P*. *falciparum* isolates with approximately 30 samples analyzed in less than two hours - including DNA extraction and data analysis. Two additional major advantages of the qHRP2/3-del assay are obvious: firstly, the ability to quantify parasitemia levels and therefore include samples based on parasitemia and secondly, to identify “masked” deletions in multiple strain infections.

The identification of *pfhrp2/3* deletions in samples with low parasitemia levels is difficult since the absence of amplification could be due to lack of sufficient template leading to incorrect reports of deletions. The conventional method depends on the successful amplification of at least two single copy reference genes to ensure sufficient template in the PCR reaction. This procedure is time-consuming and labour-intensive. The qHRP2/3-del assay uses an alternative inclusion criterion, based on the Cq value of its internal control. The pre-defined exclusion criteria of all samples that have parasitemia below 5 parasites/µL will improve the quality, reproducibility and comparability of malaria parasite survey data obtained with the qHRP2/3-del assay.

The ability to detect “masked” *pfhrp2/3* deletions is probably the most interesting feature of the qHRP2/3-del assay, because it will allow studying the epidemiology of *pfhrp2/3* deletions in malaria endemic regions with a high proportion of infections caused by multiplicity of infections, particular sub-Saharan Africa^22^. The qHRP2/3-del assay correctly identified infections that contain two strains, one with a deletion and the other one without a deletion, based on a difference in the Cq values derived from the amplification of the *pfhrp2/3* gene targets and the *pfrnr2e2* control. However, currently we cannot exclude that nucleotide sequence variations located in the binding sites of the oligonucleotides used in the PfHRP2/3-del assay could potentially also lead to variation in ΔCq values. The ΔCq application of our novel PfHRP2/3-del assay in additional studies including a larger sample size will improve our understanding of the relevance of “masked” *pfhrp2* and *pfhrp3* gene deletions and their impact on reliability of malaria RDT test results.

Two Tanzanian isolates had an increased ΔCq value for both *pfhrp* genes, indicating the presence of *pfhrp2/3* deletions in the East African nation. This was recently confirmed when *pfhrp2* and *pfhrp3* deletions were identified in Tanzania and Uganda^23^.Together with findings from Kenya^24^, the Democratic Republic of Congo^25^, Rwanda^26^ and Mozambique^27^ there is strong evidence for the existence of *pfhrp2/3* deletions in this region. Therefore, establishing programs which systematically monitor *pfhrp2/3* deletions and their impact on the performance of RDTs is advised.

## Conclusion

The qHRP2/3-del assay presented here is suitable for high-throughput screening of *P*. *falciparum* strains to identify *pfhrp2/3* gene deletions in different malaria endemic settings, including areas with high a proportion of multiple strain co-infections. With growing availability of qPCR instruments in reference laboratories in sub-Saharan countries, this assay could be used as surveillance method to monitor over time the potential expansion of *P*. *falciparum* strains carrying *pfhrp2* and *pfhrp3* deletions.

## Methods

### *P*. *falciparum* isolates from tanzania, equatorial guinea and peru

In this study a total of 205 *P*. *falciparum* isolates collected from three different malaria endemic regions, East Africa, West-Central Africa and South America were included. The samples from East Africa (n = 90) were collected in rural southern Tanzania (TZ) as part of a malaria baseline survey^28^. The West-Central African isolates (n = 47) were identified among blood donors living in Malabo, Equatorial-Guinea (EG)^18^. Both samples sets were analyzed locally, at the Bagamoyo branch of the Ifakara Health Institute and the laboratory of the Equatorial Guinea Malaria Vaccine Initiative using harmonized protocols. Briefly, genomic DNA was isolated either from 6 circles with 2 mm diameter of dried blood spots (Tanzania) or 180 µL whole blood (Equatorial Guinea) using the Quick-DNA Miniprep kits (Zymo Research, Irvine, USA). *P*. *falciparum* was identified and quantified using published qPCR protocols based on varATS^29^. Extracted DNA (n = 68) from Peruvian isolates (PE), collected between 2008–2009 and 2015–2016 around Iquitos city, was shipped to the Swiss Tropical and Public Health Institute for *pfhrp2/3* characterization by qHRP2/3-del assay.

### Additional parasite isolates and laboratory strains

Forty-nine PfNF54 isolates from Controlled Human Malaria Infections (CHMI) conducted in Bagamoyo, Tanzania (ClinicalTrials.gov: NCT02613520^30^) as well as genomic DNA isolated from 8 laboratory strains with known *pfhrp2/3* deletion status (Pf3D7, Pf7G8, PfDD2, PfHB3, PfNF135.C10, PfNF166.C8, PfNF54 and PfFC27) were used as controls. The 1st WHO International Standard *for Plasmodium falciparum* DNA Nucleic Acid Amplification Techniques (NIBSC code: 04/176, herein referred to as PfIS) was used to assess the performance of the qHRP2/3-del assay. Non-falciparum *Plasmodium* species, including *P*. *malariae (Pm)*, *P*. *ovale curtisi (Poc)*, *P*. *ovale wallikeri (Pow)*, *P*. *vivax (Pv)* and *P*. *knowelsi (Pk)* and an additional 28 samples from malaria negative individuals living in Tanzania were used to assess specificity of the assay.

### Detection of the *pfhrp2* and *pfhrp3* genes by conventional nested PCR

*P*. *falciparum* positive samples collected in Tanzania were selected for detection of *pfhrp2* and *pfhrp3* genes by nested PCR. As a reference gene, the *msp2* gene was amplified using a previously described protocol^31^. All isolates with successful *msp2* amplification were analyzed for the presence of *pfhrp2* and *pfhrp3* genes using primers spanning exon 1, the intron, and exon 2^32^. All PCR products were separated and visualized on a 2% agarose gel. Cultured parasite isolate PfDD2 (*pfhrp2* deletion) was used as a control for all nested PCR experiments on *pfhrp2* while PfHB3 (*pfhrp3* deletion) was used as a control for all nested PCR experiments on *pfhrp3*. PfNF54 (no *pfhrp2/3* deletion) was used as a positive control for both *pfhrp* genes. *Pfhrp2/3* deletion status of the Peruvian *P*. *falciparum* isolates was analyzed previously following the procedures described elsewhere^9^. Results were shared to be used for the evaluation of the qHRP2/3-del assay.

### Design of qHRP2/3-del assay

Published *pfhrp2/3* primer sequences for conventional PCR were adapted to the qPCR platform using EvaGreen® qPCR Mix (Solis BioDyne, Tartu, Estonia). The primers were tested with different DNA concentrations extracted from PfNF54, PfDD2 and PfHB3 strains, corresponding to parasitemia levels of 1 and 100 parasites/µL. The best performing primer pairs, in terms of specificity and sensitivity, were then used in combination with newly designed TaqMan® hydrolysis probes. The *pfhrp2/3* oligo sequences were systematically optimized using the trial-and-error approach. As the internal control of the qHRP2/3-del assay we amplify a *P*. *falciparum* specific 107 bp long sequence of the ribonucleotide reductase R2_e2 (*pfrnr2e2*), a distantly related paralog of the canonical eukaryotic small subunit ribonucleotide reductase R2, that is unique to apicomplexan species^14^. The performance of *pfrnr2e2* as a biomarker for detection and quantification of *P*. *falciparum* was tested by direct comparison with parasitemia levels obtained from a 18 S rDNA based qPCR assay^33^. A sensitivity of 89.1% for samples with parasitemia >1 parasite/µL and a Bland-Altman ratio of 0.99 (95% CI: −0.012–2.5) demonstrate its robustness and accuracy as internal control (Supplementary File 4). Genomic sequences for *pfrnr2e2* (PF3D7_1015800), *pfhrp2* (PF3D7_0831800) and *pfhrp3* (PF3D7_1372200) of Pf3D7 strain were obtained from PlasmoDB. A *pfhrp2/3* sequence alignment including five reference strains from West-Africa (Pf3D7, PfNF54), Guinea (PfNF166.C8), Brazil (Pf7G8) and Cambodia (PfNF135.C10) revealed no SNPs in oligo binding regions suggesting a high degree of conservation within the target region of the *pfhrp2/3* genes (Supplementary Files 2 and 3). The *pfhrp2*, *pfhrp3* and *pfrnr2e2* sequences for Pf7G8, PfNF135.C10, PfNF166.C8 and PfNF54 were obtained from whole genome sequencing^34^. The Geneious version 8.1.9 software (Biomatters Ltd, Auckland, New Zealand) was used for sequence alignments and oligo designs. Relevant information concerning the oligos used in the qHRP2/3-del assay is summarized in Table 1.

### Sample analysis with qHRP2/3-del assay

Amplification and qPCR measurements were performed using the Bio-Rad CFX96 Real-Time PCR System (Bio-Rad Laboratories, California, USA). The thermal profile used for qHRP2/3-del assay is as follows: Taq polymerase activation for 5 min at 95 °C, followed by 45 cycles of 15 s at 95 °C and 35 s at 57.5 °C. 2 µL DNA was added to 8 µL reaction master mix containing 1x Luna Universal Probe qPCR Master Mix (New England Biolabs, Ipswich, USA) and 1x qHRP2/3-del Primer Mix (Table 1). All qPCR assays were run in triplicates with appropriate controls including Non-Template Control and DNA from PfDD2, PfHB3 and PfNF54 as controls for the *pfhrp2/3* deletion status.

### Data management and statistical analysis

#### Preliminary analysis of qPCR data

Cq values were obtained from the Bio-Rad CFX96 Manager 3.1 software (Bio-Rad Laboratories, California, USA) after setting the threshold manually. Cq values were transferred and linked to the samples’ metadata using a custom-designed database for storage and analysis of qPCR data. Only samples with a Cq ≤ 40.0 for the internal control, *pfrnr2e2*, were considered eligible for analysis of *pfhrp2/3* deletion status. ΔCq were calculated by subtraction of *pfrnr2e2* Cq values from *pfhrp2* or *pfhrp3* Cq values.

#### Analytical performance of qHRP2/3-del assay and quantification of parasitemia

Based on the PfIS a serial dilution ranging from 0.01–10’000 parasites/µL was prepared and used to assess the performance of the qHRP2/3-del assay. The slope, y-intercept, qPCR efficiency and R^2^ was established for each target. The Limit of Detection (LOD) was defined as the lowest PfIS parasitemia with a positive amplification in 4 out of 6 replicates. Parasitemia was estimated using linear regression derived from serial dilution of the PfIS and the *pfrnr2e2* target which serves as the internal control of the qHRP2/3-del assay.

#### Graphical representation and statistical analysis

We used R version 3.5.1 for creating ggplot2-based graphs using the packages *ggpubr*, *gridextra* and *scales*. The Diagnostic test evaluation calculator (freely available at https://www.medcalc.org/calc/diagnostic_test.php) was used for analytical validation of qHRP2/3-del assay performance. Cohen’s kappa including 95% confidence intervals, providing a measure of agreement, was calculated using STATA version 12.0 software (Stata Corp LP; College Station, Texas, USA). P values < 0.05 were considered as significant for all statistical analysis.

### Ethical approval and informed consent

The samples analyzed in this study were collected in different studies. All studies were approved by the appropriate institutions and informed consent was obtained from all participants. The CHMI samples were collected during a clinical study, registered at Clinical Trials.gov (NCT02613520), and conducted under a U.S. FDA IND application. The study was performed in accordance with Good Clinical Practices. All samples analyzed in this publication were obtained according to the approved study protocol. The protocol was approved by the institutional review boards of the Ifakara Health Institute (IHI/IRB/No: 32–2015), and the National Institute for Medical Research Tanzania (NIMR) (NIMR/HQ/R.8a/Vol.IX/2049), by the Ethikkommission Nordwest- und Zentralschweiz, Basel, Switzerland (Ref. No. 15/104), and by the Tanzania Food and Drug Authority (Auth. No. TZ15CT013). For the Tanzanian sample collection ethics approval for the study was granted by the institutional review boards of Ifakara Health Institute (IHI/IRB/No: 18–2015) and by NIMR (NIMR/HQ/R.8a/Vol.IX/2015). For the sample collection from Equatorial Guinea approval was given by the Ministry of Health and Social Welfare. The collection, transport and storage of the blood samples from Peru was approved by the Human Ethics Committee from Universidad Peruana Cayetano Heredia (UPCH 52707 & 59751).

## Supplementary information

Supplementary file 1-4

## Data Availability

All data generated or analyzed during this study are included in this published article and its Supplementary Information Files.
