# Supplementary material for: A multiplex qPCR approach for detection of pfhrp2 and pfhrp3 gene deletions in multiple strain infections of Plasmodium falciparum
Source: Sci Rep. 2019 Sep 11;9:13107. doi: 10.1038/s41598-019-49389-2 (PMC6739368; doi:10.1038/s41598-019-49389-2)

A multiplex qPCR approach for detection of *pfhrp2* and *pfhrp3* gene deletions in multiple strain infections of *Plasmodium falciparum*

Tobias Schindler, Anna C. Deal, Martina Fink, Etienne Guirou, Kara A. Moser, Solomon M. Mwakasungula, Michael G. Mihayo, Said A. Jongo, Prosper P. Chaki, Salim Abdulla, Paulo C. Manrique Valverde, Katherine Torres, Jose R. Bijeri, Joana C. Silva, Stephen L. Hoffman, Dionicia Gamboa, Marcel Tanner and Claudia Daubenberger

Supplementary file 1. Sequence alignment (first 350 bp) of pfhrp2 and pfhrp3 genes including intron sequence (grey). qHRP2/3-del primer (black) and probe (green) binding regions are highlighted. Nucleotide sequence differences in oligo binding regions between *pfhrp2* and *pfhrp3* are indicated in red.

```
pfhrp2            1 ATGGTTTCCTTCTCAAAAAATAAAGTATTATCCGCTGCCGTTTTTGCCTC     50
                    ||||||||||||||||||||||||.||||||||||||||||||||||.||
pfhrp3            1 ATGGTTTCCTTCTCAAAAAATAAAATATTATCCGCTGCCGTTTTTGCTTC     50

pfhrp2           51 CGTACTTTTGTTAGATAACGTAAGCATTTTAATTGCAAATAGAAATAAAT    100
                    ||||||||||||||||||||||||.||||||||||||||||.||||||||
pfhrp3           51 CGTACTTTTGTTAGATAACGTAAGTATTTTAATTGCAAATATAAATAAAT    100

pfhrp2          101 ATATCATTCAATTATAAAAATACATATAATAATAATATATATATATATAT    150
                    |||.||.|.|.|||||||||||||||||||||||.|||||||||||||||
pfhrp3          101 ATAACACTTACTTATAAAAATACATATAATAATATTATATATATATATAT    150

pfhrp2          151 ATATATTTATATAT-----TGTATATATATAAATTTTTTCATTTTTAAAT    195
                    ||||||.|||||||     |.|||||||.||.||||||.|||||||||||
pfhrp3          151 ATATATATATATATATATATATATATATATGTACATTTTTACATTTTTAAAT    200

pfhrp2          196 GCTTTTTTATTTTTATATAGAATAATTCCGCATTTAATAATAACTTGTGT    245
                    |.|||||.|||||  ||||||||||.||||.||||||.||||||||||.|
pfhrp3          201 GATTTTTCATTTT--TATAGAATAACTCCGAATTTAACAATAACTTGTTT    248

pfhrp2          246 AGCAAAAATGCAAAAGGACTTAATTTAAATAAGAGATTATTACACGAAAC    295
                    |||||||||||||||||||||||||.|||||||||||||||||||||||.
pfhrp3          249 AGCAAAAATGCAAAAGGACTTAATTCAAATAAGAGATTATTACACGAAAG    298

pfhrp2          296 TCAAGCACATGTAGATGATGCCCATCATGCTCATCATGTAGCCGATGCCC    345
                    |||||||||||.||.|||||||||||||||.|||||||||||.|||||||
pfhrp3          299 TCAAGCACATGCAGGTGATGCCCATCATGCACATCATGTAGCTGATGCCC    348
```

Supplementary file 2. Sequence alignment (first 360 bp) of *pfhrp2* for five reference strains from Africa (3D7, NF54, NF166.C8), Brazil (7G8) and Cambodia (NF135.C10). The oligo binding sites are highlighted in grey.

```
NF135      ATGGTTTCCTTCTCAAAAAATAAAGTATTATCCGCTGCCGTTTTTGCCTCCGTACTTTTG    60
3D7        ATGGTTTCCTTCTCAAAAAATAAAGTATTATCCGCTGCCGTTTTTGCCTCCGTACTTTTG    60
7G8        ATGGTTTCCTTCTCAAAAAATAAAGTATTATCCGCTGCCGTTTTTGCCTCCGTACTTTTG    60
NF54       ATGGTTTCCTTCTCAAAAAATAAAGTATTATCCGCTGCCGTTTTTGCCTCCGTACTTTTG    60
NF166      ATGGTTTCCTTCTCAAAAAATAAAGTATTATCCGCTGCCGTTTTTGCCTCCGTACTTTTG    60
           ************************************************************

NF135      TTAGATAACGTAAGCATTTTAATTGCAAATAGAAATAAATATATCATTCAATTATAAAAA    120
3D7        TTAGATAACGTAAGCATTTTAATTGCAAATAGAAATAAATATATCATTCAATTATAAAAA    120
7G8        TTAGATAACGTAAGCATTTTAATTGCAAATAGAAATAAATATATCATTCAATTATAAAAA    120
NF54       TTAGATAACGTAAGCATTTTAATTGCAAATAGAAATAAATATATCATTCAATTATAAAAA    120
NF166      TTAGATAACGTAAGCATTTTAATTGCAAATAGAAATAAATATATCATTCAATTATAAAAA    120
           ************************************************************

NF135      TACATATAATAATAATATATATATATATATATATATTTATATATTGTATATATATAAATT    180
3D7        TACATATAATAATAATATATATATATATATATATATTTATATATTGTATATATATAAATT    180
7G8        TACATATAATAATAATATATATATATATATATATATTTATATATTGTATATATATAAATT    180
NF54       TACATATAATAATAATATATATATATATATATATATTTATATATTGTATATATATAAATT    180
NF166      TACATATAATAATAATATATATATATATATATATATTTATATATTGTATATATATAAATT    180
           ************************************************************

NF135      TTTTCATTTTTAAATGCTTTTTTATTTTTATATAGAATAATTCCGCATTTAATAATAACT    240
3D7        TTTTCATTTTTAAATGCTTTTTTATTTTTATATAGAATAATTCCGCATTTAATAATAACT    240
7G8        TTTTCATTTTTAAATGCTTTTTTATTTTTATATAGAATAATTCCGCATTTAATAATAACT    240
NF54       TTTTCATTTTTAAATGCTTTTTTATTTTTATATAGAATAATTCCGCATTTAATAATAACT    240
NF166      TTTTCATTTTTAAATGCTTTTTTATTTTTATATAGAATAATTCCGCATTTAATAATAACT    240
           ************************************************************

NF135      TGTGTAGCAAAAATGCAAAAGGACTTAATTTAAATAAGAGATTATTACACGAAACTCAAG    300
3D7        TGTGTAGCAAAAATGCAAAAGGACTTAATTTAAATAAGAGATTATTACACGAAACTCAAG    300
7G8        TGTGTAGCAAAAATGCAAAAGGACTTAATTTAAATAAGAGATTATTACACGAAACTCAAG    300
NF54       TGTGTAGCAAAAATGCAAAAGGACTTAATTTAAATAAGAGATTATTACACGAAACTCAAG    300
NF166      TGTGTAGCAAAAATGCAAAAGGACTTAATTTAAATAAGAGATTATTACACGAAACTCAAG    300
           ************************************************************

NF135      CACATGTAGATGATGCCCATCATGCTCATCATGTAGCCGATGCCCATCATGCTCATCATG    360
3D7        CACATGTAGATGATGCCCATCATGCTCATCATGTAGCCGATGCCCATCATGCTCATCATG    360
7G8        CACATGTAGATGATGCCCATCATGCTCATCATGTAGCCGATGCCCATCATGCTCATCATG    360
NF54       CACATGTAGATGATGCCCATCATGCTCATCATGTAGCCGATGCCCATCATGCTCATCATG    360
NF166      CACATGTAGATGATGCCCATCATGCTCATCATGTAGCCGATGCCCATCATGCTCATCATG    360
           ************************************************************
```

Supplementary file 3. Sequence alignment (first 360 bp) of *pfhrp3* for five reference strains from Africa (3D7, NF54, NF166.C8), Brazil (7G8) and Cambodia (NF135.C10). The oligo binding sites are highlighted in grey.

```
NF166      ATGGTTTCCTTCTCAAAAAATAAAATATTATCCGCTGCCGTTTTTGCTTCCGTACTTTTG     60
NF135      ATGGTTTCCTTCTCAAAAAATAAAATATTATCCGCTGCCGTTTTTGCTTCCGTACTTTTG     60
3D7        ATGGTTTCCTTCTCAAAAAATAAAATATTATCCGCTGCCGTTTTTGCTTCCGTACTTTTG     60
7G8        ATGGTTTCCTTCTCAAAAAATAAAATATTATCCGCTGCCGTTTTTGCTTCCGTACTTTTG     60
NF54       ATGGTTTCCTTCTCAAAAAATAAAATATTATCCGCTGCCGTTTTTGCTTCCGTACTTTTG     60
           ************************************************************

NF166      TTAGATAACGTAAGTATTTTAATTGCAAATATAAATAAATATAACACTTACTTATAAAAA     120
NF135      TTAGATAACGTAAGTATTTTAATTGCAAATATAAATAAATATAACACTTACTTATAAAAA     120
3D7        TTAGATAACGTAAGTATTTTAATTGCAAATATAAATAAATATAACACTTACTTATAAAAA     120
7G8        TTAGATAACGTAAGTATTTTAATTGCAAATATAAATAAATATAACACTTACTTATAAAAA     120
NF54       TTAGATAACGTAAGTATTTTAATTGCAAATATAAATAAATATAACACTTACTTATAAAAA     120
           ************************************************************

NF166      TACATATAATAATATTATATATATATATATATATATATATATATATATATATATATATGT     180
NF135      TACATATAATAATATTATATATATATATATATATATATATATATATATATATATATATGT     180
3D7        TACATATAATAATATTATATATATATATATATATATATATATATATATATATATATATGT     180
7G8        TACATATAATAATATTATATATATATATATATATATATATATATATATATATATATATGT     180
NF54       TACATATAATAATATTATATATATATATATATATATATATATATATATATATATATATGT     180
           ************************************************************

NF166      ACATTTTTACATTTTTAAATGATTTTTCATTTTTATAGAATAACTCCGAATTTAACAATA     240
NF135      ACATTTTTACATTTTTAAATGATTTTTCATTTTTATAGAATAACTCCGAATTTAACAATA     240
3D7        ACATTTTTACATTTTTAAATGATTTTTCATTTTTATAGAATAACTCCGAATTTAACAATA     240
7G8        ACATTTTTACATTTTTAAATGATTTTTCATTTTTATAGAATAACTCCGAATTTAACAATA     240
NF54       ACATTTTTACATTTTTAAATGATTTTTCATTTTTATAGAATAACTCCGAATTTAACAATA     240
           ************************************************************

NF166      ACTTGTTTAGCAAAAATGCAAAAGGACTTAATTCAAATAAGAGATTATTACACGAAAGTC     300
NF135      ACTTGTTTAGCAAAAATGCAAAAGGACTTAATTCAAATAAGAGATTATTACACGAAAGTC     300
3D7        ACTTGTTTAGCAAAAATGCAAAAGGACTTAATTCAAATAAGAGATTATTACACGAAAGTC     300
7G8        ACTTGTTTAGCAAAAATGCAAAAGGACTTAATTCAAATAAGAGATTATTACACGAAAGTC     300
NF54       ACTTGTTTAGCAAAAATGCAAAAGGACTTAATTCAAATAAGAGATTATTACACGAAAGTC     300
           ************************************************************

NF166      AAGCACATGCAGGTGATGCCCATCATGCACATCATGTAGCTGATGCCCATCATGCACATC     360
NF135      AAGCACATGCAGGTGATGCCCATCATGCACATCATGTAGCTGATGCCCATCATGCACATC     360
3D7        AAGCACATGCAGGTGATGCCCATCATGCACATCATGTAGCTGATGCCCATCATGCACATC     360
7G8        AAGCACATGCAGGTGATGCCCATCATGCACATCATGTAGCTGATGCCCATCATGCACATC     360
NF54       AAGCACATGCAGGTGATGCCCATCATGCACATCATGTAGCTGATGCCCATCATGCACATC     360
           ************************************************************
```

Supplementary file 4. Evaluation of *pfrnr2e2* as a biomarker for detection and quantification of *P. falciparum* parasites during controlled human malaria infection (CHMI). Bland-Altman plot of average parasitaemia density (x-axis) and ratio of parasitaemia levels calculated between internal control of qHRP2/3-del assay, pfrnr2e2, and qPCR assays based on 18S rDNA detection (y-axis). Average ratio (black line) and 95 % limits of agreement (dashed line) are depicted.

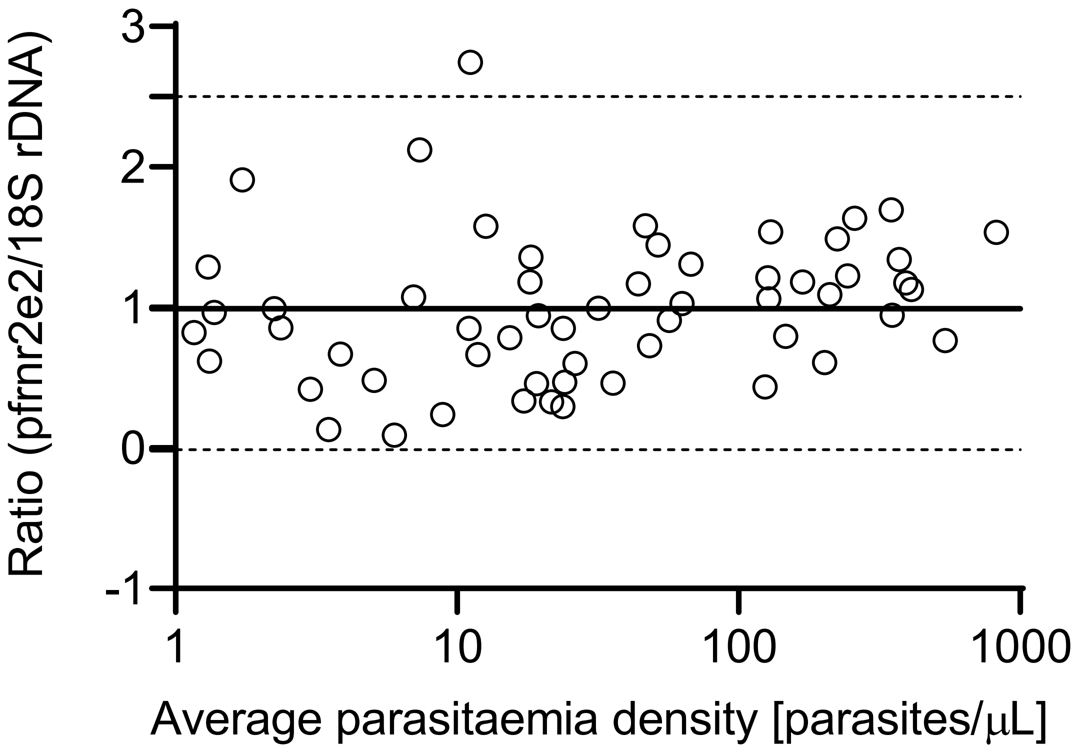

Supplement: Supplementary file 1 — 4 [file 41598_2019_49389_MOESM1_ESM.pdf]
